# Effects of implementation strategies aimed at improving high-value verification methods of nasogastric tube placement: A systematic review

**DOI:** 10.3389/fnut.2022.1009666

**Published:** 2022-12-01

**Authors:** Jiamin Li, Xiangyu Sun, Xinjuan Wu

**Affiliations:** Department of Nursing, Chinese Academy of Medical Sciences and Peking Union Medical College, Peking Union Medical College Hospital, Beijing, China

**Keywords:** implementation strategies, high-value, verification methods, nasogastric tube placement, systematic review

## Abstract

**Background:**

X-ray and pH testing, which clinical practice guidelines have proven to be effective in determining nasogastric tube (NGT) placement, were named the high-value methods. Implementation strategies can help to integrate high-value methods into particular contexts. The aim of this systematic review was to summarize the evidence of implementation strategies aimed at improving high-value verification methods of NGT placement.

**Methods:**

PubMed, ProQuest, and CINAHL were searched until June 2022. The Cochrane Effective Practice and Organization of Care (EPOC) taxonomy was used to categorize implementation strategies.

**Results:**

The initial search identified 1,623 records. Of these, 64 full-text studies were reviewed. Finally, 12 studies were included and used for qualitative synthesis. Eleven studies used an education component as an implementation strategy. Only one study based their implementation strategy on a barriers and facilitators assessment. None of the studies reported enough detail of the implementation strategy used in their studies. Seven studies were eligible for inclusion in the meta-analysis. Three of these seven studies revealed a significant improvement of the high-value method after strategy implementation. As heterogeneity was present in the high level, the pooled effect estimated was not calculated.

**Conclusion:**

Most studies used an implementation strategy with an educational component. Unfortunately, no conclusion can be drawn about which strategy is most effective for improving high-value verification methods of NGT placement due to a high level of heterogeneity and a lack of studies. We recommend that future studies fully connect their implementation strategies to influencing factors and better report the details of implementation strategies.

**Systematic review registration:**

[www.crd.york.ac.uk/PROSPERO/], identifier [CRD42022349997].

## Introduction

Nasogastric tubes (NGT) are widely used to deliver food to patients. Over 790,000 NGT are used in the National Health Service (NHS) each year (1). Most of the time, nurses at the beside insert these tubes blindly, while this is generally considered a reasonably harmless practice, it is not. During the insertion procedures, the NGT can easily misplace into the respiratory tract, often leading to serious consequences if the misplace is not recognized before feedings are provided. These problems tend to occur, especially in patients undergoing sedation and intubation, when the cough reflex has been abolished (2), with a high incidence of complications during NGT positioning (3). According to the NHS report (1), in an approximate 5-year period, there were 95 cases of feed being injected into the respiratory tract. The patient is mentioned as having died in 32 of these cases, although this is not always obvious if the death was directly connected, considering that several patients were critically ill before the NGT was inserted.

To avoid serious events of NGT being inadvertently positioned, it is necessary to determine NGT placement. Methods to identify NGT placement mainly include radiography, aspirate pH, auscultation and aspirate appearance. However, several guidelines (4–6) state that auscultation methods should be avoided completely because air introduced *via* the tube can be heard in a number of bodily regions. It is insufficient to distinct between NGT location in the stomach and respiratory tracts. Observing aspirate appearance as a sign of correct NGT placement is against by guidelines (1, 5, 6) because the appearance of aspirates from the stomach and respiratory tract appear to overlap. These methods, which have been shown to be ineffective, can potentially hurt patients and waste precious resources, were named low-value care (7). While a method in which evidence has proven to be effective and beneficial to patients is called high-value care (8). There is general consensus among all guidelines that an X-ray, when correctly taken and read, is the most precise method for differentiating between the placement of an NGT in the gastric and respiratory tract (9). Aspirate pH is a distinct difference between fasting gastric juice and respiratory tract aspirates. Compared to X-ray, pH testing of aspirates is cost-effective. Several guidelines (1, 6) suggest that aspirate pH can be used as the first-line test to identify NGT placement. Thus, X-ray and aspirate pH, which are suggested by evidence, are high-value verification methods of NGT placement.

However, high-value verification methods of NGT placement are not always used in practice. Recent surveys showed that only 11% of critical nurses use the pH method to identify NGT placement (10); only 26.9% of nurses chose X-ray as the gold standard (11). Implementation research can help to address the challenge between high-value care and clinical practice. Implementation strategies are central to implementation research, which is defined as “the study of strategies to integrate evidence-based interventions into particular contexts” (12). Including the particular means or methods for adopting and maintaining evidence-based intervention (13), e.g., education, reminders, audit, and feedback. These implementation strategies should be determined by an assessment of the barriers and facilitators that impact high-value care because it is considered to promote the compliance, acceptance, and effectiveness of these implementation strategies (14, 15). Descriptions of implementation strategies (e.g., actor, action, action goal, and dosage) must be specific and clear enough to support repeatability in both research and practice, similar to all intervention studies (16). To improve the high-value verification methods of NGT placement, many implementation strategies have been performed in some studies (17, 18). However, an understanding of implementation strategies aimed at improving high-value verification methods of NGT placement and the effectiveness of these different strategies is lacking. Therefore, the purpose of this systematic review is to summarize the evidence of implementation strategies that have been used to improve high-value verification methods of NGT placement.

## Methods

This systematic review was conducted according to the Preferred Reporting Items for Systematic Reviews and Meta-analyses (PRISMA) statement. This systematic review protocol was registered in PROSPERO (number: CRD42022349997). The PICO (population, intervention, comparison, and outcome) used to guide this review was as follows. P: the medical worker who determined NGT placement; I: implementation strategy aimed to improve high-value verification methods of NGT placement; C: without an implementation strategy; and O: effect on the volume of the high-value verification method of NGT placement.

### Search strategies

To find all relevant studies that focus on implementation strategies aiming to improve high-value verification methods of NGT placement, a systematic literature search was performed in the PubMed, ProQuest, and CINAHL electronic databases. The search strategies were tailored to the characteristics of each database using the following medical subject headings (MeSH) or keywords as search terms: “enteral nutrition,” “nasogastric,” “tube,” “intubate,” “pH,” “X-ray,” “radiograph,” “evidence-based practice,” “quality improvement,” and “implement.” The search was restricted to the studies released until June 2022. Other search filter restrictions were not implemented. An expert health librarian guided the search. An example PubMed search is as follows: ((“enteral nutrition”[Mesh]) OR (“enteral nutrition”[TI/AB]) OR (“nasogastric”[TI/AB])) AND ((“tube*”[TI/AB]) OR (“intubat*”[TI/AB])) AND ((“pH”[TI/AB]) OR (“X-ray*”[TI/AB]) OR (“radiograph”[TI/AB])) AND ((“evidence-based practice”[Mesh]) OR (“evidence-based practice”[TI/AB]) OR (“implement*”[TI/AB]) OR (“quality improvement”[Mesh]) OR (“quality improvement” [TI/AB])).

### Selection of studies

All search results were imported into Endnote (version X9). Two authors (A and B) independently screened the titles and abstracts after eliminating duplicates. For relevant records, full-text versions of manuscripts were acquired and screened. In these processes, disparities that could not be resolved by discussion between the two reviewers were resolved with the help of the third author (C). The high-value verification method of NGT placement was defined as a method that has been proven effective and benefits the patient by evidence. In this review, high-value verification methods of NGT placement refer to pH testing and X-ray. The following criteria were used to determine study eligibility in this systematic review.

a) Study type: Any study that includes a control group, such as randomized controlled trial (RCT), cluster RCT, quasi-RCT, non-RCT, before-after study.
b) Setting: Hospitals, community settings, long-term care facilities, and nursing homes.
c) Outcome: The study had to report on the effect of the implementation strategy on the volume of the high-value verification method of NGT placement.
d) Language: English language articles.

Animal studies, letters, case studies, and editorials were not included. The meta-analysis included studies with available data.

### Quality evaluation

The quality of the studies was assessed using “the Joanna Briggs Institute Meta-Analysis of Statistics Assessment and Review Instrument standardized critical appraisal instrument (JBI MAStARI) for quasi-experimental studies” (19) by two independent authors (A and B). This instrument is made up of nine items: cause effect clear, participants similar, similar care other than intervention, control group, multiple measurements, follow-up, standardized measurement, reliable measurement, and statistical analysis. Those items can be rated yes, no, unclear, or not applicable. A point is given if the item is selected as “yes.” Disparities in the scores were addressed by consensus between two reviewers or by discussion with a third researcher (C).

### Data extraction

Data from the included studies were extracted by one researcher (A) using a self-designed standardized data extraction tool. A second researcher (B) verified the extracted data independently. Any disagreements were settled by discussion among the researchers until agreement was obtained. If this was not attainable, a third researcher (C) made a decision based on the information provided. Information about country, design, type of high-value method, implementation strategy, barriers and facilitators assessment, target population, and outcome was collected from all included studies. The outcome refers to the change in volume of the high-value verification method of NGT placement (e.g., use rate, score).

The implementation strategies were categorized using the “Cochrane Effective Practice and Organization of Care (EPOC)” taxonomy. The EPOC taxonomy includes implementation strategies, governance arrangements, financial arrangements, and delivery arrangements 4 main domains and more than 100 subcategories, such as education, reminders, organizational culture, audit and feedback.

### Statistical analysis

The information from all included studies was extracted and summarized to present a descriptive and narrative synthesis of the overall evidence of implementation strategies aimed at improving the high-value verification method of NGT placement.

The results are shown in forest plots created with Review Manager 5.4 to help evaluate the efficacy of implementation strategies. Mantel-Haenszel’s random effects model was used to pool the data, and relative risk (RR) and 95% confidence intervals were computed. To assess the degree of heterogeneity among the included studies, the *I*^2^ statistics of Higgins were utilized. We could see the results as indicating a moderate to high level of heterogeneity when the *I*^2^ was 50% or above. Subgroup analyses were carried out in cases of heterogeneity. Subgroup analyses were conducted based on the type of implementation strategy (type of strategy according to EPOC taxonomy and single vs. multifaceted) and type of high-value verification method of NGT placement (X or pH). We conducted subgroup analysis for the type of implementation strategy to determine which strategy was more advanced. We conducted subgroup analysis for the type of high-value verification method of NGT placement since the factors (e.g., supporting evidence, credibility, and feasibility) of the implemented high-value method might influence the effectiveness of the implementation strategy. A subgroup analysis was only carried out when at least two studies with, respectively, the same implementation strategy or same high-value method could be included in each subgroup. When *I*^2^ > 50% after subgroup analysis, the pooled effect estimate was not performed. When very few studies for a similar result were found, funnel plot analysis, which helps to explore the problem of publication bias, would not be carried out.

## Results

### Search and screening results

**FIGURE 1:**
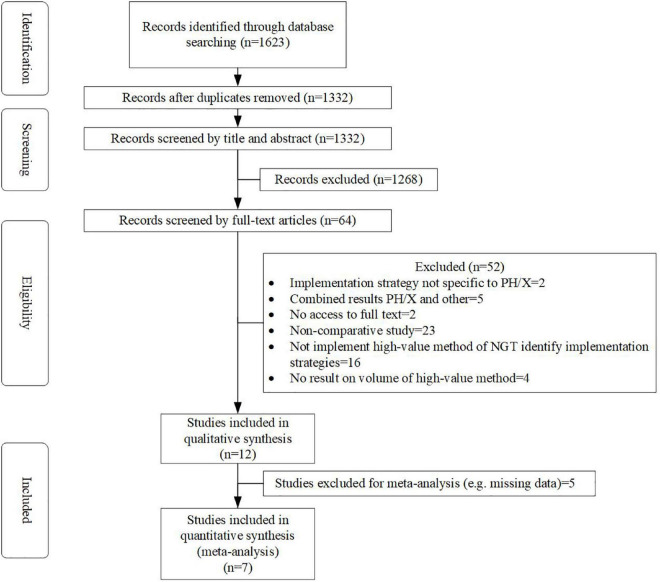
Flowchart of study selection.

Electronic databases yielded 1,623 records, and 291 duplicates were then deleted. Sixty-four full-text studies were obtained and screened after the titles and abstracts were screened, and 52 were excluded. Finally, 12 articles (17, 18, 20–29) were included and used for qualitative synthesis in this review (Figure 1). The excluded articles were due to (1) studies that did not have a reference group (*n* = 23), (2) studies that did not include implementation strategies to improve high-value verification methods of NGT placement (*n* = 16), and (3) other (e.g., full text was not available, no outcomes showed on the volume of the high-value method, combined results of pH/X and other methods) (*n* = 13).

### Quality of the included studies

Table 1 shows the risk of bias of the 12 included studies evaluated by the JBI MAStARI tool. Generally, the included studies were moderate quality. All studies obtained a score ranging from 5 to 8 (in a whole range of 0–9). Three studies obtained a score of 5 (23, 25, 28). Two studies obtained a score of 6 (20, 27). Five studies obtained a score of 7 (17, 21, 22, 24, 29). Two studies obtained a score of 8 (18, 26). Most studies did not obtain a high score, which was primarily the result of a low score on the “establishment control group” and “outcome measured in a reliable way” items.

**TABLE 1:** Results of critical appraisal (*n* = 12).

References	Q1	Q2	Q3	Q4	Q5	Q6	Q7	Q8	Q9	Score
Kisting et al. (17)	Yes	Yes	Yes	No	Yes	Yes	Yes	Unclear	Yes	7
Roe et al. (24)	Yes	Yes	Yes	No	Yes	Yes	Yes	Unclear	Yes	7
Guerrero et al. (18)	Yes	Yes	Yes	No	Yes	Yes	Yes	Yes	Yes	8
Cole (20)	Yes	Unclear	Yes	Yes	No	Yes	Yes	Unclear	Yes	6
Law et al. (23)	Yes	Yes	Yes	No	No	Not applicable	Yes	Unclear	Yes	5
Tho et al. (21)	Yes	Yes	Yes	No	Yes	Yes	Yes	Unclear	Yes	7
Richardson et al. (22)	No	Yes	Yes	Yes	No	Yes	Yes	Yes	Yes	7
Taylor et al. (29)	Yes	Yes	Yes	No	Yes	Yes	Yes	Unclear	Yes	7
Lee et al. (27)	Yes	Yes	Yes	No	Yes	Not applicable	Yes	Unclear	Yes	6
Farrington et al. (28)	Yes	Yes	Yes	No	No	Unclear	Yes	Unclear	Yes	5
Kenny and Goodman (25)	Yes	Unclear	Yes	No	Yes	Not applicable	Yes	Unclear	Yes	5
Yang et al. (26)	Yes	Yes	Unclear	Yes	Yes	Yes	Yes	Yes	Yes	8

### Characteristics of the included studies

In this review, the 12 included studies were published between 2006 and 2019 (Table 2). All included studies used a quasi-experimental design. Of these, eleven studies used a before-after design. Most studies were performed in the United States (USA) (*n* = 4) (17, 22, 25, 28) and the United Kingdom (UK) (*n* = 4) (20, 23, 24, 27). Of the 12 included studies, three focused their implementation strategy on improving pH testing (17, 26, 29), three on improving X-ray (23–25), and six on improving these two methods (18, 20–22, 27, 28). Of the 9 studies (17, 18, 20–22, 26–29) that used pH testing, four used a pH level of 5.5 as the cutoff point (20, 26, 27, 29), three used a pH level of 5 as the cutoff point (17, 18, 22), one used a pH value of 4 as the cutoff point (28), and one used a pH value of 6 as the cutoff point (21). The implementation strategy used was directed at nurses (*n* = 7) (17, 18, 21, 22, 25, 26, 28), doctors (*n* = 2) (23, 27), radiographers (*n* = 1) (24), and multidisciplinary staff (*n* = 2) (20, 29).

**TABLE 2:** Characteristics of the included studies (*n* = 12).

References	Country	Design	Type of high-value method	Implementation strategy (sorted by EPOC taxonomy)	Barriers and facilitators identified (Yes/No)	Target group	Outcome	Before	After	Difference	Statistical analyses (Yes/No)	*P* ≤ 0.05 (Yes/No)
Kisting et al. (17)	USA	Before-after	pH	Education; audit and feedback; local opinion leaders; health information system	No	Nurses	The use rate of pH	8/71 (11.3%)	59/64 (92.2%)	80.9%	No	–
Roe et al. (24)	UK	Before-after	X	Education	No	Radiographers	The rate of confidence in image interpretation	58/98 (59.2%)	96/98 (98.0%)	38.8%	Yes	Yes
Guerrero et al. (18)	Spain	Before-after	pH; X	Education	No	Nurses	The use rate of pH	42/553 (7.6%)	133/245 (54.3%)	46.7%	Yes	Yes
Cole (20)	UK	Before-after	pH or X	Education; reminders; health information system; packages of care	No	Nurses, doctors	The use rate of pH/X	4/13 (31%)	9/12 (75%)	44.0%	No	–
Law et al. (23)	UK	Before-after	X	Education; organizational culture; audit and feedback; health information system; packages of care	No	Doctors	The accuracy of image interpretation	185/192 (96%)	199/200 (99.5%)	3.5%	No	–
Tho et al. (21)	Singapore	Before-after	pH; X	Education; local consensus processes; clinical practice guidelines; audit and feedback; packages of care	No	Nurses	The use rate of pH (X for special cases)	22/26 (84.6%)	40/46 (87%)	2.4%	No	–
Richardson et al. (22)	USA	Before-after	pH; X	Audit and feedback; continuous quality improvement; interprofessional education; local consensus processes; managerial supervision; stakeholder involvement in policy decisions	No	Nurses	The use rate of pH (X for special cases)	12/15 (80%)	20/20 (100%)	20%	No	–
Taylor et al. (29)*	Australia	Before-after	pH	Education; organizational culture; reminders; tailored intervention; staffing models; community mobilization; packages of care	Yes	Nurses, doctors, dieticians	The use rate of pH as first-line (Median)	11%	60%	49%	No	–
Lee et al. (27)*	UK	Before-after	pH; X	Education; reminders	No	Doctors	The accuracy of knowledge	3%	33%	30%	No	–
Farrington et al. (28)*	USA	Before-after	pH; X	Education; audit and feedback; health information system; local opinion leaders; reminders; communities of practice; monitoring the performance of the delivery of healthcare	No	Nurses	The use rate of pH	18%	52.8%	34.8%	No	–
Kenny and Goodman (25)*	USA	Before-after	X	Education; reminders	No	Nurses	Knowledge score (Mean, standard deviation)	0.62 (0.48)	0.71 (0.46)	0.09	Yes	No
Yang et al. (26)*	China	Non-RCT	pH	Education, audit and feedback; reminders; local consensus processes	No	Nurses	Knowledge score (Mean)	4.1	5.2	1.1	No	–

*Result not used for meta-analysis.

### Strategies to improve high-value methods

Ten of 12 included studies had multifaceted implementation strategies (17, 20–23, 25–29) (Table 2). The other two studies had a single implementation strategy (18, 24), which indicates that there was only one strategy component found in the implementation strategies. A total of 18 kinds of implementation strategies were used by the included studies. Education (materials, meetings, and games) is the most commonly used strategy. Eleven of the 12 included studies used an education component as an implementation strategy (17, 18, 20, 21, 23–29). Half of the studies used audit and feedback as an implementation strategy (17, 21–23, 26, 28). Health information systems, reminders, and packages of care strategies were used separately by one-third of the included studies. Local consensus processes and organizational culture were used separately by three and two studies. The other 11 kinds of implementation strategies were used by only one study. However, only one of 12 included studies based their implementation strategy on barriers and facilitators assessment (29). Additionally, none of the included studies reported enough detail of each implementation strategy used in the studies. Relatively, details of education (meetings, materials, games, and outreach visits) strategy get the clearest reported than other strategies. There are three studies (18, 21, 24) have clearly described the period of the education strategy. It differed from 30 to 90 min. Other details (e.g., actor, action, justification) of the education strategy were not described.

Different outcome measurements of implementation strategy were used by the included studies (Table 2), which included the use rate of pH/X (*n* = 7) (17, 18, 20–22, 28, 29), the accuracy of knowledge (*n* = 1) (27), the accuracy of image interpretation (*n* = 1) (23), the rate of confidence in image interpretation (*n* = 1) (24) and knowledge score (*n* = 2) (25, 26). Outcome measurements about contextual factors (e.g., cost, appropriateness) were not found in these studies. The improvement in volume of the high-value method ranged from 2.4 (21) to 80.9% (17) (*n* = 10) and from a score of 0.09 (25) to 1.1 (26) (*n* = 2). Three studies (18, 24, 25) performed statistical analysis; of these, two studies (18, 24) had a positive significant effect on the volume of the high-value method. These two positive significant studies (18, 24) had a single implementation strategy.

### Effectiveness of implementation strategies (meta-analysis)

Seven (17, 18, 20–24) of the 12 included studies were eligible for inclusion in the meta-analysis (Figure 1). Four studies (26–29) were excluded because of missing data, and one study (25) was excluded because outcomes could not be calculated with other studies (Table 2).

**FIGURE 2:**
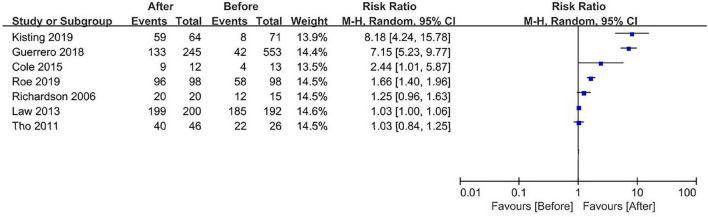
Forest plot for the effect of the implementation strategy.

Seven studies (17, 18, 20–24) included for meta-analysis used the rate of the high-value method as outcome measurements. A forest plot is used to display the data and computed RR (Figure 2). Considerable heterogeneity was present in the high level among the seven studies. Subgroup analyses were performed for the type of implementation strategy and type of high-value verification method of NGT placement. As heterogeneity was still large after subgroup analyses, we did not calculate a pooled effect estimated. Of seven studies (17, 18, 20–24), three studies (17, 18, 24) revealed a significant improvement in the high-value method after strategy implementation. The RR for these three studies was 8.18 [95% CI 4.24, 15.78] (17), 7.15 [95% CI 5.23, 9.77] (18), and 1.66 [95% CI 1.40, 1.96] (24), respectively. The other four studies showed a nonsignificant small (20) or no improvement (21–23) of the high-value NGT placement verification method.

## Discussion

To the best of our knowledge, the current study is the first systematic review on implementation strategies for high-value verification methods of NGT placement. This review identifies X-ray and pH as high-value verification methods of NGT placement based on current evidence (1, 6, 9). As research progresses, a future device capable of combining the presence of two separate methods (such as CO_2_ and pH) could have higher diagnostic accuracy in identifying the correct positioning of NGT (30). This “combined” method may substitute for pH testing and become a high-value confirmation method of NGT placement in the future.

The majority of the included studies were conducted in the USA and the UK. The reason may be that the problem of NGT misplacement has prompted high concern by these two countries. For example, NGT misplacement has been classified as a warning incident by the Joint Commission of Healthcare Organizations in the USA (31). Similarly, NGT misplacement was identified as one of the original eight “never events” by the National Patient Safety Agency (NASP) in the UK (32).

The design of almost all studies was quasi-experimental. The goal of implementation science is to increase the adoption, application, and sustainability of effective healthcare practices by physicians, hospitals, and systems (33). Politicians or managers may be unwilling in certain implementation science settings to have a portion of engaged patients or locations randomized to a controlled group, particularly for high-profile or high-urgency clinical situations. In these circumstances, quasi-experimental designs enable implementation scientists to perform robust analyses, although the type of study design has intrinsic limitations (33). To give academics increasingly reliable and practical methods for responding to important implementation science questions, design innovations are still needed.

The included studies used different pH values as the cutoff point to determine NGT placement. Several studies have been published in the literature investigating the diagnostic accuracy of different pH cutoff points to distinguish between gastric and other placement (30, 34). However, there is still much indecision regarding the best cutoff point for pH at the present time (9). Moreover, the pH method has some limitations; for example, gastric acid inhibiting medications (such as proton pump inhibitors) elevate gastric pH and make it more difficult to determine NGT placement on the basis of pH testing (9). Therefore, even though the pH method has several advantages, such as reducing the need for costly X-ray, and being easy to apply, the use of this method should be cautious about identifying the pH cutoff level in clinical practice.

The educational component (materials, meetings, and games) is the most common implementation strategy among almost all studies. This is similar to other systematic reviews of implementation studies (35). However, given that both studies with a significant beneficial effect and studies without an effect contained an educational component, there can be no direct correlation between the incorporation of education components and successful implementation. Multifaceted implementation strategies were not more effective than a single implementation strategy in this review. Two studies (18, 24) had a single implementation strategy among three studies (17, 18, 24), which revealed a significant improvement in the high-value method after strategy implementation. This may be because multiple strategies can be mutually exclusive. More research focusing on optimal combinations and interactions of implementation strategies is needed.

Using an implementation strategy based on barriers and facilitators assessment was recommended to boost the efficacy of implementation strategies (14, 15). However, the results show that only one study (29) included in this review assessed the barriers and facilitators prior to creating the implementation strategy, and the study (29) showed a 50% improvement of the high-value identification method (no statistical testing). Ineffective implementation strategies in this review may have been caused by a lack of assessment of barriers and facilitators impacting the implementation of the high-value verification methods of NGT placement. Future implementation studies should consider the determinants when determining an implementation strategy.

Additionally, none of the studies included sufficient details on implementation strategy. Specification limitations raise important issues that hinder repeatability in both practice and research. Similar to all intervention research, the implementation strategies must be thoroughly and accurately stated, such as action target, dose and temporality (16). There are some relevant guidelines that might help authors (16, 36, 37) describe and report implementation strategies.

The interstudy heterogeneity hindered the meta-analysis in our review, which is consistent with other systematic reviews addressing the effectiveness of implementation strategies to change healthcare (38). The included studies varied mainly in terms of implementation strategies, target populations, outcome indicators and countries. More studies that explore the effect of implementation strategies aimed at improving high-value verification methods of NGT placement are needed. Implementation also necessitates consideration of a variety of critical contextual issues, such as service system and provider attitudes. Implementation outcomes also need to include provider attitudes (acceptability) and contextual factors (penetration, appropriateness, cost) (39). Therefore, to assist in explaining the mechanisms and causal links within implementation processes and advance an evidence base around successful implementation, future research should also concentrate on analyzing additional outcomes in addition to the efficacy of implementation strategies (39).

### Strengths and limitations

The search strategies in the current review were designed with the help of an experienced librarian. This ensured that the search strategies were professional, comprehensive, and effective. However, some relevant articles were likely to have been overlooked because of language or publication restrictions. In addition, due to missing data, not all studies could be included in the meta-analysis.

## Conclusion

Most studies used the educational component as an implementation strategy. However, no conclusion can be drawn on the most effective implementation strategy for improving high-value verification methods of NGT placement because of a lack of studies and the high level of heterogeneity. Future research is required to determine whether implementation strategies are more successful for implementation if they completely link their strategy to the barriers and facilitators. To enable reproducibility in both practice and research, the details of implementation strategies need to be reported.

## Data availability statement

The original contributions presented in this study are included in the article/supplementary material, further inquiries can be directed to the corresponding author.

## Author contributions

XW: conception, study design, execution, acquisition of data, analysis and interpretation, and substantially revised or critically reviewed the article. JL: conception, study design, execution, acquisition of data, analysis and interpretation, and drafted or written. XS: study design, execution, acquisition of data, and analysis and interpretation. All authors contributed to data analysis, drafting or revising the article, agreed on the journal to which the article will be submitted, gave final approval of the version to be published, and agreed to be accountable for all aspects of the work.
